# Effect of specimen type on free immunoglobulin light chains analysis on the Roche Diagnostics cobas 8000 analyzer

**DOI:** 10.1186/s40064-015-1546-x

**Published:** 2015-12-08

**Authors:** Louis S. Nelson, Bryan Steussy, Cory S. Morris, Matthew D. Krasowski

**Affiliations:** Department of Pathology, University of Iowa Hospitals and Clinics, Iowa City, IA 52242 USA

**Keywords:** Immunoglobulin light chains, Laboratory automation, Nephelometry, Plasma, Serum

## Abstract

The measurement of free immunoglobulin light chains is typically performed on serum; however, the use of alternative specimen types has potential benefits. Using the Freelite™ kappa and lambda free light chains assay on a Roche Diagnostics cobas 8000 c502 analyzer, we compared three specimen types (serum, EDTA-plasma and lithium heparin plasma separator gel-plasma) on 100 patients. Using Deming regression and eliminating outliers (limiting data to light chain concentrations below 400 mg/L), the three specimen types showed comparable results for kappa light chain concentration, lambda light chain concentration, and kappa/lambda ratio with slopes close to 1.0 and y-intercepts close to zero. EDTA-plasma showed slightly more positive bias relative to serum than lithium heparin. Analysis using EDTA-plasma and lithium heparin plasma showed comparable linearity, precision, and temperature stability. A single sample showing hook effect (not in the comparison set) gave comparable results using either plasma specimen type. For the Freelite™ kappa and lambda free light chains assay, both EDTA-plasma or lithium heparin-plasma can serve as acceptable substitutes for serum, at least for the Roche cobas 8000 analyzer.

## Background

Measurement of kappa and lambda free immunoglobulin light chains in serum has been shown to be valuable in the diagnosis and management of a variety of diseases, especially plasma cell disorders such as multiple myeloma, Waldenström’s macroglobulinemia, AL amyloidosis, and light chain deposition diseases (Bradwell et al. 2001; Dimopoulos et al. 2011; Dispenzieri et al. 2009, 2010; Hoedemakers et al. 2011; Katzmann et al. 2005; Lachmann et al. 2003; Morris et al. 2007; Snozek et al. 2008; Tosi et al. 2013). Serum free light chain analysis is often used in conjunction with serum and urine protein electrophoresis (Hoedemakers et al. 2011; Kim et al. 2014; McTaggart et al. 2013). Serum is the mandatory specimen for protein electrophoresis; thus, the same serum specimen is often used for measurement of free light chains. However, analysis of plasma may have potential practical advantages compared to serum. For example, the ability to use plasma as a specimen for free light chain analysis may limit number of blood collection tubes needed during phlebotomy for some patients (e.g., if plasma but not serum is needed for other tests co-ordered for a patient) or to allow add-on orders for free light chain analysis if serum is not available as a pre-existing specimen (Nelson et al. 2015). The ability to run free light chain analysis on automated chemistry instrumentation typically allows for faster turnaround time than protein electrophoresis, which requires more specialized instrumentation and result interpretation.

In this study we compared the differences between serum and plasma for measurement of kappa and lambda free light chains using the Freelite™ serum free light chain assays on a Roche Diagnostics cobas 8000 c502 analyzer. Plasma specimens obtained from ethylenediaminetetraacetic acid (EDTA)-anticoagulated tubes and lithium heparin plasma separator tubes (PST) were used for the comparisons. A previous study has compared plasma versus serum for another marketed free light chain assay (N Latex FLC) and showed similar results using either specimen type (te Velthuis et al. 2011). Another study compared serum versus serum separator gel and lithium heparin plasma samples for the Freelite™ assay on a Dade Behring BNII analyzer (Hansen et al. 2012). However, there is no published study doing the same comparison for the Freelite™ assay on the Roche cobas system, and the manufacturer instructions for the Freelite™ assay on this analytical platform only list serum as the acceptable specimen type (Freelite™ Human Kappa Free and Human Lambda Free Light Chains package insert.).

## Experimental

### Sample collection and processing for comparison studies

This study had approval from the University of Iowa Institutional Review Board (protocol #201407792). Testing was performed in the University of Iowa Hospitals and Clinics (UIHC) core clinical laboratory. The layout and informatics of this clinical laboratory has been detailed in previous publications (Krasowski et al. 2014, 2015). The inclusion criteria were: (1) patient who had free light chain analysis performed on a serum specimen, (2) EDTA-anticoagulated and lithium heparin PST specimens drawn on patient for clinical testing during same phlebotomy encounter, and (3) sufficient specimen remaining in the EDTA and PST specimens for light chain analysis after performance of provider-ordered clinical testing. The details on the three specimen types were: BD Vacutainer^®^ red top silica clot activator coated tube (BD Diagnostics, Franklin Lakes, NJ), BD Vacutainer^®^ light green top plasma separator tubes (PST™) containing polymer gel and lithium heparin (BD Diagnostics), and BD Vacutainer^®^ pink top spray coated K_2_EDTA tube (BD Diagnostics). No extra tubes were drawn on any patient for purposes of this study, i.e., all analyses used pre-existing specimens leftover from clinical testing that would otherwise have been discarded.

Upon completion of the clinically ordered tests, the specimens were transferred to a refrigerator for storage using a Roche Diagnostics (Indianapolis, IN) P701 automated archival retrieval system (Nelson et al. 2015). Samples were stored for up to 16 h until they were retrieved for use in the study. When samples were retrieved, they were centrifuged and loaded on to the Roche Diagnostics cobas 8000 Modular Analytics System c502 analyzer and assayed using the Freelite™ kappa and lambda free light chains assay (Freelite™ Human Kappa Free and Human Lambda free light chains package insert 2001).

Following the package insert procedure, kappa light chain measurements for serum specimens are linear to 56.25 mg/L. Values that exceed 56.25 mg/L are treated with ×10 dilution with saline. Values that still exceed linearity require a manual ×21 dilution with saline. Lambda light chain measurements are linear to 93.33 mg/L. Similar to the procedure with kappa light chains, lambda light chain values that exceed 93.33 mg/L are treated with ×10 dilution with saline. Values that still exceed linearity require a manual ×21 dilution with saline.

### Linearity studies

Linearity was determined according to CLSI guideline EP6A (Clinical and Laboratory Standards Institute 2003) using plasma samples just above the upper measuring range for serum, i.e., 56.25 mg/L for kappa and 93.33 mg/L for lambda. At least 10 dilutions of 90–2.5 % were measured for both EDTA-plasma and lithium heparin PST matrices. Five replicates for each dilution were measured. The mean result was analyzed by linear and cubic analysis. Fits were evaluated using the Microsoft Excel add-in Analyze-it^®^.

### Method imprecision

The precision study was performed according to CLSI EP5-A2 guideline (Clinical and Laboratory Standards Institute 2004). Plasma pools were made from routine patient samples that had no detectable monoclonal bands with serum electrophoresis and immunotyping.

### Reference ranges and medical decision levels

The normal (reference) ranges for the free light chains following manufacturer recommendations in the package insert are: kappa (3.30–19.40 mg/L), lambda (5.71–26.30 mg/L), kappa/lambda ratio (0.26–1.65). The lower and upper limits of the reference ranges for serum in the assay package insert were considered the medical decision levels (MDL). Assay measurement using serum (specimen type recommended in package insert) was considered the gold standard.

### Statistical analysis

Linear regression and statistical analysis was performed using EP Evaluator release 11 (Data Innovations, Inc., South Burlington, VT). Deming linear regression was performed. Identification of outliers used an algorithm in EP Evaluator that identifies points whose distance from the regression line exceeds 10 times the standard error of estimate (SEE), where SEE is computed from the data set with outliers excluded.

## Results

### Precision studies

**Fig. 1:**
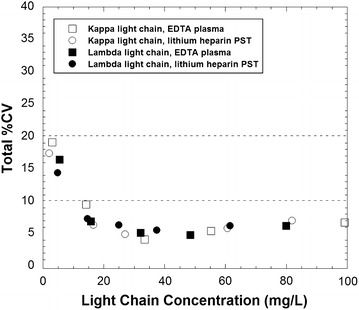
Precision profile of the total coefficient of variation (CV) estimated according to CLSI EP5-A2. *Squares* (*open* and *filled*) indicate kappa and lambda concentrations, respectively, using EDTA-plasma. *Circles* (*open* and *filled*) indicate kappa and lambda concentrations, respectively, using lithium heparin plasma separator tubes

The precision of the kappa and lambda light chain assays at different levels of control material are summarized in Table 1. The coefficient of variation (CV) was less than 5 % for the within- and between-run precision studies. The results of the precision studies for plasma pools are summarized in Fig. 1. The  % CV was generally 5–7 % or less across most of the measuring range except for kappa and lambda concentrations less than 5 mg/L. At concentrations near 1 mg/L, the  % CV values approach 20 %.

**Table 1:** Precision using two different levels of control material

Light chain assay	Mean (mg/L)	Within-run imprecision % CV (n = 10)	Between-run imprecision % CV (n = 20)
Kappa	17.4	2.9	4.8
Kappa	34.1	3.5	4.6
Lambda	30.0	3.4	4.9
Lamdba	63.3	2.6	4.5

*CV* coefficient of variation

### Linearity studies

Serial dilutions of samples with a concentration just above the measuring range (56.25 mg/L for kappa and 93.33 mg/L for lambda) were prepared. For kappa, linearity was confirmed between 1.0 and 56.25 mg/L for both EDTA-plasma and lithium heparin PST (maximum difference between linear and cubic fit of 18.8 %). For lambda, linearity was confirmed between 0.8 and 93.33 mg/L (maximum difference between linear and cubic fit of 17.4 %).

### Method comparison

**Fig. 2:**
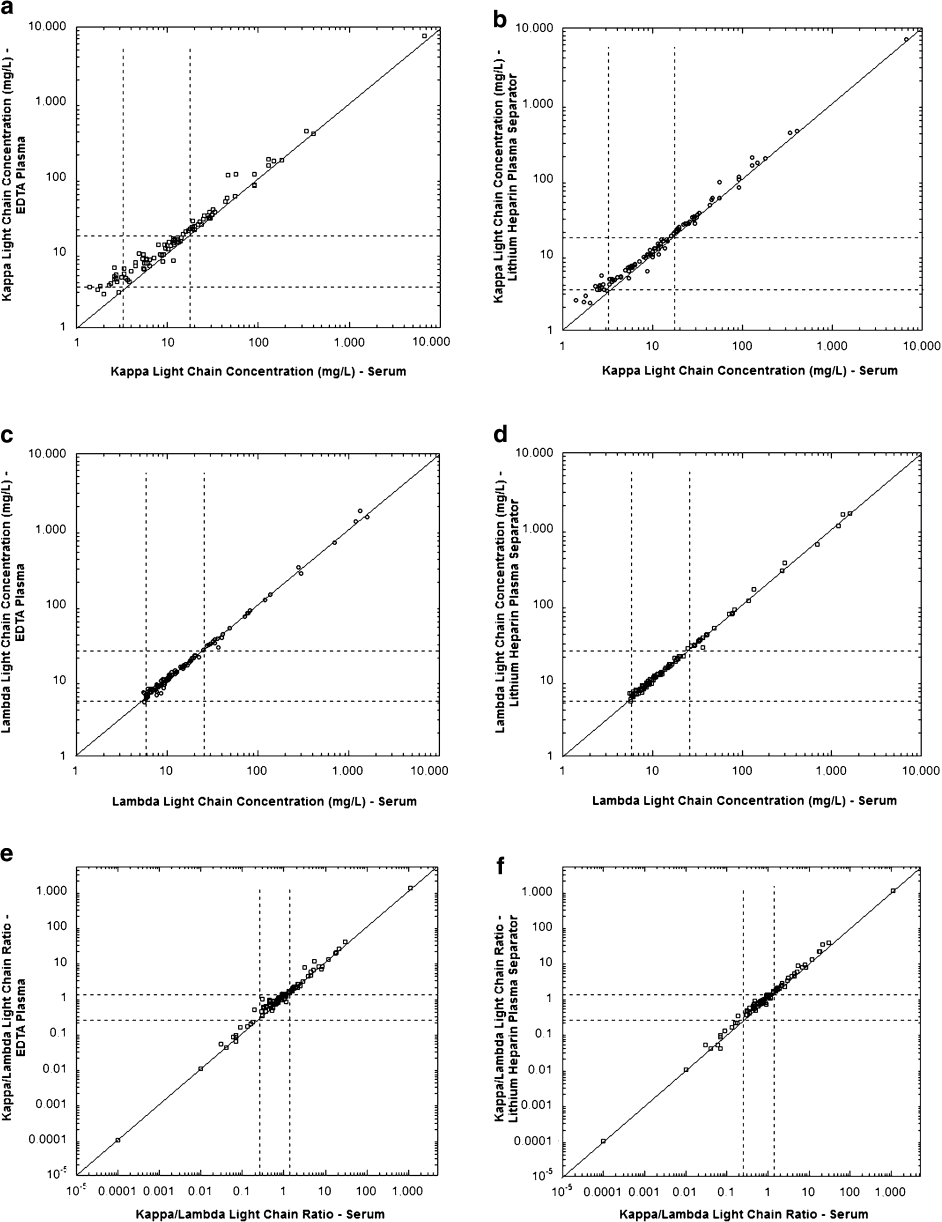
Comparisons of serum versus EDTA-plasma or lithium heparin-plasma separator tube as specimens for measurement of free light chains (n = 100). The *graphs* are scatter *plots* of data for kappa concentration (**a**, **b**), lambda concentration (**c**, **d**), and kappa/lambda ratio (**e**, **f**). The *line* is the line of identity

**Fig. 3:**
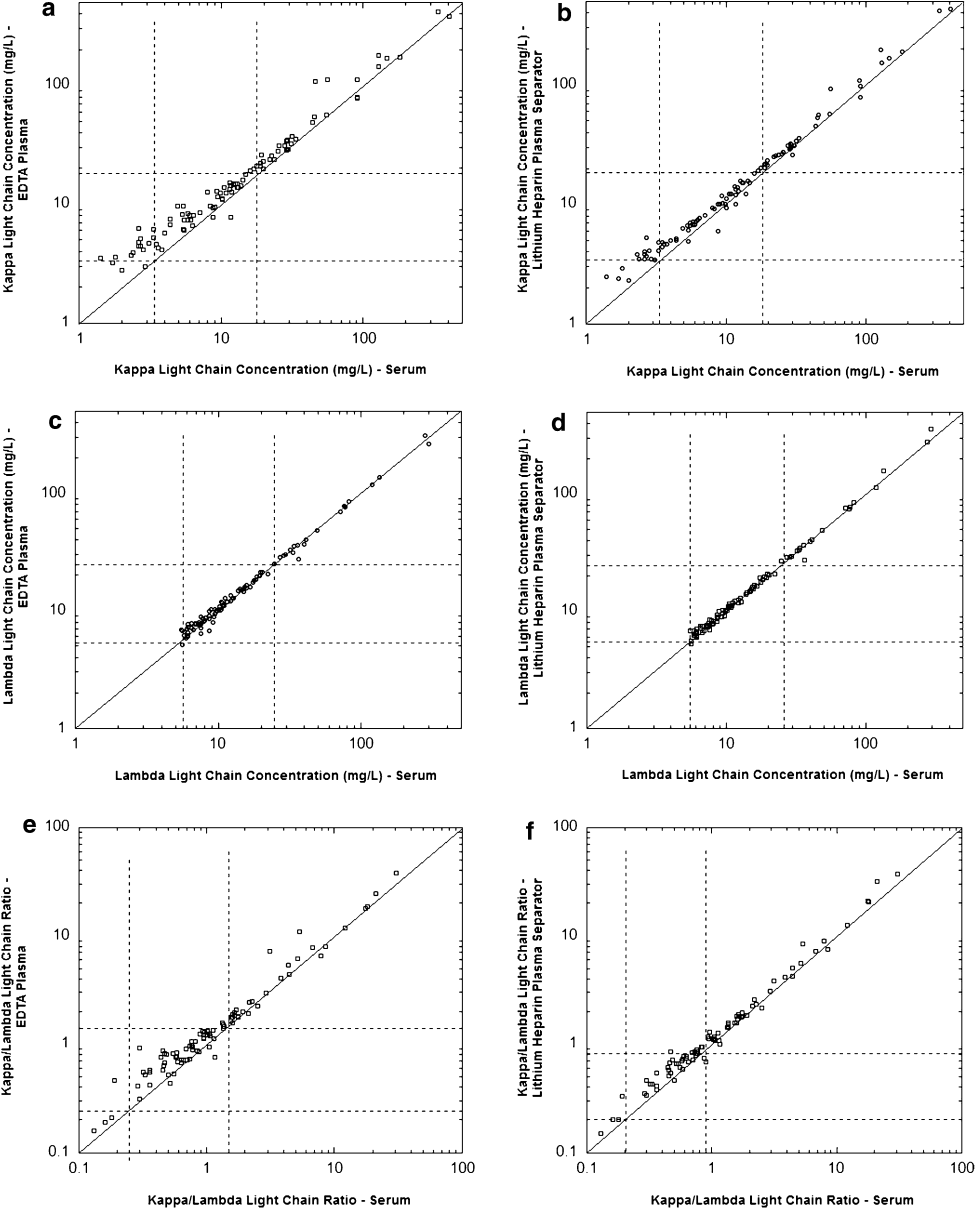
Comparisons of serum versus EDTA-plasma or lithium heparin-plasma separator tube as specimens for measurement of free light chains showing subset of data. The *graphs* are scatter *plots* of data for kappa concentration (**a**, **b**; restricted to 400 mg/L or less), lambda concentration (**c**, **d**; restricted to 400 mg/L or less), and kappa/lambda ratio (**e**, **f**; restricted to 100 or less). The *line* is the line of identity

Samples from a total of 100 patients met the inclusion criteria for this study with sufficient serum, EDTA-plasma, and lithium heparin-plasma for free light chain analysis. Scatterplots for kappa, lambda and kappa/lambda ratio are shown for all 100 samples in Fig. 2 and for the subset of kappa and lambda values less than 400 mg/L in Fig. 3. By classifying the specimens on the basis of the reference intervals, there was 84 and 86 % agreement, respectively, between serum and either EDTA-plasma or lithium heparin PST specimens for the kappa light chain assay (Table 2). The agreement rate was 95 % for the lambda light chain assays (serum versus either EDTA-plasma and lithium heparin plasma) and 98 % for the kappa/lambda ratio (serum versus either EDTA-plasma and lithium heparin plasma) (Table 2). All of the discrepancies with respect to reference interval were the result of the plasma result being higher than the value obtained from serum (Table 2). A summary of the specimens showing discrepancies is in Table 3, with clinical history and age/gender of patient described.

**Table 2:** Concordance tables between plasma and serum for light chain analysis

Assay	Comparison	Identical %	Serum below ref. range/plasma within ref. range	Serum within ref. range/plasma above ref. range
Kappa	*EDTA vs. serum*	84	11	5
Kappa	*Lithium heparin PST vs. serum*	86	10	4
Lambda	*EDTA vs. serum*	98	2	0
Lambda	*Lithium heparin PST vs. serum*	98	1	1
Kappa/lambda ratio	*EDTA vs. serum*	95	1	4
Kappa/lambda ratio	*Lithium heparin PST vs. serum*	95	1	4

**Table 3:** Samples with discrepancy with respect to reference intervals

Patient age, gender, and clinical history	Serum			EDTA-plasma			Lithium heparin PST		
	Kappa (mg/L)	Lambda (mg/L)	K/L	Kappa (mg/L)	Lambda (mg/L)	K/L	Kappa (mg/L)	Lambda (mg/L)	K/L
Discrepancy involving kappa									
60 Y M, multiple myeloma, IgG kappa	2.6	5.9	0.44	4.4	5.8	0.76	3.8	6.2	0.61
66 Y F, multiple myeloma, IgG lambda	2.7	5.8	0.47	4.4	6.6	0.67	3.7	6.8	0.54
44 Y F, multiple myeloma, IgG lambda	17.8	40.0	0.45	20.8	36.8	0.57	20.2	39.5	0.51
60 Y F, multiple myeloma, IgG lambda	18.8	10.7	1.76	20.8	11.6	1.79	20.1	11.1	1.81
61 Y M, multiple myeloma, IgG kappa	2.8	6.1	0.46	4.1	6.5	0.63	4.1	6.1	0.67
51 Y M, multiple myeloma, IgG kappa	2.7	5.8	0.47	5.1	6.2	0.82	5.3	6.2	0.85
86 Y F, multiple myeloma, lambda light chain	2.3	76	0.03	3.7	77.5	0.05	3.8	75.1	0.05
60 Y F, multiple myeloma, IgA lambda	2.4	6.7	0.36	3.9	6.9	0.57	3.5	6.5	0.54
70 Y F, multiple myeloma, IgA lambda	2.6	8.1	0.32	4.8	8.7	0.55	3.5	8.2	0.43
39 Y M, multiple myeloma, IgG lambda	2.6	8.6	0.30	6.3	6.8	0.93	4	8.7	0.46
60 Y M, hairy cell leukemia	19.1	18.7	1.02	26.1	19.6	1.33	21.8	19.7	1.11
62 Y M, biclonal IgG kappa + lambda light chain^a^	3.1	1195	<0.01	4.7	1275	<0.01	3.4	1103	<0.01
66 Y F, multiple myeloma, lambda light chain^a^	17.0	296	0.06	19.7	263	0.08	19.3	359	0.05
Discrepancy involving kappa and lambda									
53 Y F, multiple myeloma, IgG kappa	18.9	24.7	0.77	21.9	25.0	0.88	21.9	26.6	0.82
84 Y M, multiple myeloma, lamba light chain	2.9	5.6	0.52	3.0	6.8	0.44	3.5	5.3	0.66
51 Y M, multiple myeloma, IgG kappa	1.8	5.5	0.33	3.6	6.9	0.52	2.9	6.8	0.43
Discrepancy involving kappa and kappa/lambda ratio									
54 Y F, multiple myeloma, kappa light chain	1.4	7.2	0.19	3.5	7.6	0.46	2.5	7.6	0.33
Discrepancy involving kappa/lambda ratio									
67 Y M, multiple myeloma, IgG kappa	29.1	18.3	1.59	34.4	18.4	1.87	29.3	18.5	1.58
64 Y M, multiple myeloma, kappa light chain	21.9	13.8	1.59	23.7	14.9	1.59	25.1	13.9	1.81
57 Y F, multiple myeloma, IgG lambda	25.1	15.7	1.60	27.7	15.6	1.78	26.2	15.5	1.69
55 Y M, multiple myeloma, lambda light chain	12.1	7.4	1.64	14.8	7.5	1.97	13.9	7.4	1.88
59 Y M, Waldenstroms, IgM lambda	19.8	12.6	1.57	22.7	12.7	1.79	23.5	12.9	1.82

*PST* plasma separator tube

^a^Also identified as outlier for lambda light chain analysis by Deming regression (Table 4)

Comparison between serum and the two plasma specimen types was done by Deming linear regression. Outliers identified in the analysis were predominantly due to specimens with kappa and lambda concentrations exceeding 400 mg/L (thus Fig. 3 is restricted to kappa and lambda concentrations less than 400 mg/L). The outliers are summarized in Table 4 (some of these overlapped with the specimens described in Table 3).

**Table 4:** Outliers identified by Deming regression analysis

Patient age, gender, and clinical history	Serum			EDTA-plasma			Lithium heparin PST		
	Kappa (mg/L)	Lambda (mg/L)	K/L	Kappa (mg/L)	Lambda (mg/L)	K/L	Kappa (mg/L)	Lambda (mg/L)	K/L
Kappa outliers									
60 Y M, multiple myeloma, kappa light chain	46.1	14.7	3.14	107	15.0	7.16	55.8	14.7	3.80
59 Y F, multiple myeloma, IgG kappa	6684	6.1	1096	7764	6.1	1273	7125	6.7	1063
52 Y M, multiple myeloma, IgG kappa	337	11.0	30.6	419	11.1	37.8	415	11.2	37.1
60 Y M, multiple myeloma, IgG kappa	56.2	10.5	5.35	112	10.3	10.87	91.9	10.8	8.51
59 Y M, multiple myeloma, IgG kappa	128	6.1	21.03	177	7.2	24.6	197	6.2	31.8
Lambda outliers									
60 Y M, plasma cell leukemia, IgG lambda	4.0	693	0.01	5.7	672	0.01	5.0	621	0.01
74 Y F, multiple myeloma, IgG lambda	4.4	1590	0.00	6.7	1457	0.00	5.2	1571	0.00
55 Y F, multiple myeloma, lambda light chain	11.8	1334	0.01	12.7	1747	0.01	12.0	1562	0.01
65 Y M, multiple myeloma, lambda light chain	9.9	278	0.04	12.5	311	0.04	10.1	278	0.04
59 Y F, acute renal failure, seropositive rheumatoid arthritis	29.8	36.4	0.82	28.8	27.2	1.06	25.9	27.4	0.95
62 Y M, biclonal IgG kappa + lambda light chain	3.1	1195	<0.01	4.7	1275	<0.01	3.4	1103	<0.01
66 Y F, multiple myeloma, lambda light chain	17.0	296	0.06	19.7	263	0.08	19.3	359	0.05

*PST* plasma separator tube

Linear regression parameters are summarized in Table 5. For all comparisons, slopes are close to 1.0. A slightly more noticeable positive bias was noted with EDTA-plasma versus serum. For lithium heparin PST versus serum, the confidence intervals for the slope and y-intercept for kappa light chain, lambda light chain, and kappa/lambda ratio overlapped with 1.000 and 0.0, respectively. At the MDL, the confidence intervals overlapped with that for serum.

**Table 5:** Linear regression summary statistics of specimen comparisons^a^

	Slope (95 % CI)^b^	Y-intercept (95 % CI)^b^ (mg/L)	Correlation coefficient	95 % CI at lower MDL^c^ (mg/L)	95 % CI at upper MDL^c^ (mg/L)
EDTA vs. serum					
Kappa	0.969 (0.950–0.988)	2.58 (1.54–3.64)	0.9954	4.8–6.8	20.4–22.4
Lambda	0.995 (0.986–1.003)	0.367 (0.127–0.608)	0.9992	5.8–6.3	26.3–26.7
κ/λ ratio	1.001 (0.982–1.021)	0.133 (0.066–0.199)	0.9957	0.33–0.46	1.73–1.84
Li-heparin plasma separator tube vs. serum					
Kappa	1.081 (1.063–1.100)	0.64 (−0.38 to 1.67)	0.9964	3.2–5.2	19.4–22.5
Lambda	0.988 (0.981–1.000)	0.279 (−0.101 to 0.557)	1.0000	5.7–6.3	26.1–26.6
κ/λ ratio	1.175 (1.154–1.196)	−0.091 (−0.188 to 0.007)	0.9961	0.12–0.31	1.65–1.83

^a^Analysis uses Deming regression excluding outliers (see Table 4)

^b^ *CI* confidence interval

^c^ *MDL* medical decision limit: kappa, 3.3 and 19.4 mg/L; lambda, 5.7 and 26.3 mg/L; κ/λ ratio, 0.26 and 1.65

### Stability studies

**Fig. 4:**
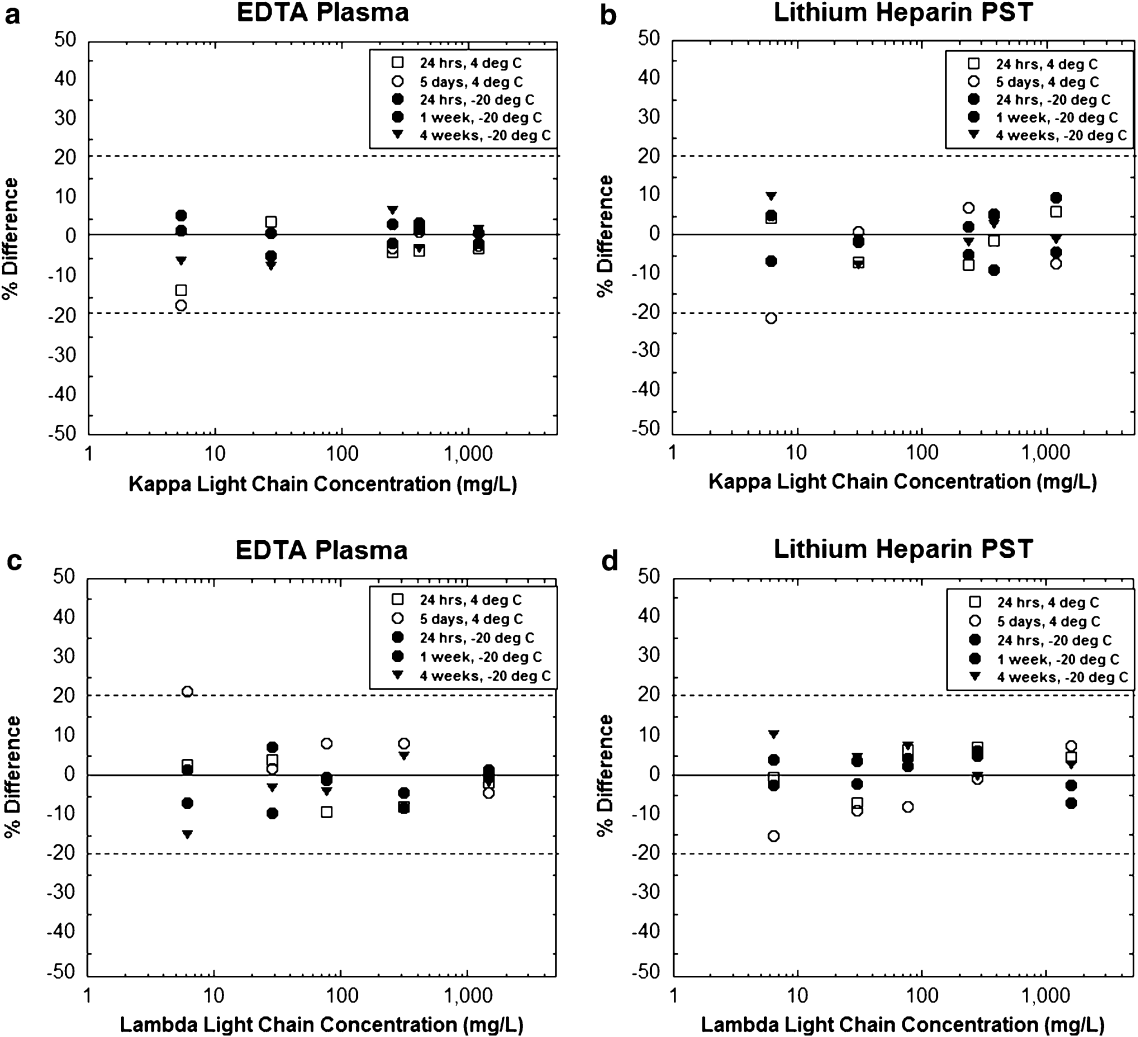
Stability of free light chain measurements at different storage conditions. The *plot* shows the percent change relative to initial analysis for kappa (**a**, **b**) and lambda (**c**, **d**) measurements using EDTA-plasma and lithium heparin plasma separator tube (PST) specimens

Stability studies were performed for pooled plasma samples stored either refrigerated (4 °C) or frozen at −20 °C. Results were generally within 10 % of those obtained at initial measurement (Fig. 4). The highest variability was seen at kappa and lambda concentrations less than 10 mg/L, similar to that described above for the precision studies (Fig. 1).

### Antigen excess

During the time period of study, a single specimen was analyzed that showed marked hook effect for lambda light chain. This specimen was from a patient whose specimens had previously shown hook effect during multiple occasions. Specimens from this patient were not in the comparison studies (occurred after those studies completed). The hook effect was comparable in EDTA-plasma and lithium heparin PST specimen. In particular, the apparent lambda light chain concentration in undiluted specimens was 89.3 and 89.8 mg/L, respectively, for EDTA-plasma and lithium heparin PST specimens. Dilution analysis shown the actual lambda concentrations to be 4053 and 3862 mg/L, respectively, in these two sample types.

## Discussion

Overall, EDTA-plasma and lithium heparin-plasma gave comparable results to serum for kappa light chain concentration, lambda light chain concentration, and kappa/lambda ratio for the Freelite™ assays performed on the cobas 8000 c502 analyzer. Similar to previous reports (Hansen et al. 2012; te Velthuis et al. 2011), these data suggest that plasma is an acceptable specimen for free light chain analysis. The precision, linearity, and analyte stability in plasma is also comparable to that described for serum in previous publications (Altinier et al. 2013; Briand et al. 2010; Cha et al. 2014; Hansen et al. 2012; Pretorius et al. 2012; Tate et al. 2003, 2009; Vercammen et al. 2015).

The highest variability seen in our study was with very low or very high kappa and lambda concentrations. This was evident in the regression outlier analysis. The light chain analysis procedure in the package insert for the Freelite™ assay on the cobas 8000 analyzers requires two dilutions (one on-line and one manual) for the highest concentrations (kappa greater than 563 mg/L and lambda greater than 933 mg/L). These dilutions offer potential for error. At high concentrations of free light chains, antigen excess is also possible (Bosmann et al. 2010; Vercammen et al. 2015). We did not study antigen excess in detail but did observe comparable results with both plasma specimen types in a patient previously observed to have hook effect with lambda concentrations. Specimens with very low values of kappa or lambda can also lead to higher variability in the kappa/lambda ratio, especially with the high imprecision typical of analyses in these low concentration ranges (Altinier et al. 2013; Briand et al. 2010; Cha et al. 2014; Hansen et al. 2012; Pretorius et al. 2012; Tate et al. 2003, 2009; Vercammen et al. 2015).

While plasma gave comparable results to serum in our study, it is probably prudent to use a single specimen type and analyzer platform for patient analysis. Reference intervals and medical decision levels should be reassessed carefully for any specimen type other than serum. The trending of light chain values is used for treatment decisions and consistency in analysis is important. Future studies can focus on different instrument platforms and assay formats. A larger set of samples can also facilitate detailed studies on how plasma compares with serum with respect to antigen excess.
